# Tree Maladaptation Under Mid-Latitude Early Spring Warming and Late Cold Spell: Implications for Assisted Migration

**DOI:** 10.3389/fpls.2022.920852

**Published:** 2022-07-06

**Authors:** Lahcen Benomar, Jean Bousquet, Martin Perron, Jean Beaulieu, Mebarek Lamara

**Affiliations:** ^1^Institut de Recherche sur les Forêts, Université du Québec en Abitibi-Témiscamingue, Rouyn-Noranda, QC, Canada; ^2^Centre d’Étude de la Forêt et Institut de Biologie Intégrative et des Systèmes, Faculté de Foresterie, de Géographie et de Géomatique, Université Laval, Québec, QC, Canada; ^3^Direction de la Recherche Forestiére, Ministére des Forêts, de la Faune et des Parcs, Québec, QC, Canada

**Keywords:** climate change and instability, assisted population migration, late-spring frost, phenology, seed transfer modeling, shoot growth cancelation, white spruce (*Picea glauca* [Moench] Voss)

## Abstract

Global warming is predicted to extend the growing season of trees and plants, and advance spring phenology. However, intensification of extreme climate events in mid-latitude forests, from weakening of the jet stream and atmospheric blockings, may expose trees to increased risk associated with more frequent late-spring frosts. Still, little is known regarding the intraspecific variation in frost tolerance and how it may be shaped by local adaptation to the climate of seed origin. As part of an assisted migration trial located in different bioclimatic zones in the province of Quebec, Canada, and following an extensive late-spring frost that occurred at the end of May 2021, we evaluated the frost damages on various white spruce (*Picea glauca*) seed sources tested on three sites (south, central, and north). The severity of frost damages was assessed on 5,376 trees after the cold spell and an early spring warming which advanced bud flush by approximately 10 days on average. The frost damage rate was similar among sites and seed sources and averaged 99.8%. Frost damage severity was unrelated to the latitude of seed origin but was variable among sites. The proportion of severely damaged trees was higher in the northern site, followed by central and southern sites. The proportion of severely damaged trees was linearly and inversely related to tree height before the frost event. Apical growth cancelation was not significantly different among seed sources including local ones, and averaged 74, 46, and 22%, respectively, in central, northern, and southern plantation sites. This study provides recommendations to limit the loss of plantation productivity associated with such a succession of spring climate anomalies. Implications for seed transfer models in the context of climate change and productivity of spruce plantations are discussed in the light of lack of local adaptation to such pronounced climate instability and ensuing large-scale maladaptation.

## Introduction

Northern forest ecosystems are expected to experience an increase in average daily temperature of at least 2.5°C by 2050 according to the International Panel on Climate Change intermediate scenario SSP2-4.5 (IPCC, 2021), and an increase in frequency, timing and duration of extreme climate events such as drought, frost, and heatwaves (Cook et al., 2018; IPCC, 2021). Seasonal climate anomalies and extreme weather events can be caused by atmospheric blockings (atmospheric pressure patterns with little movement), which are resulting from persistent waveness of the jet stream in mid and high northern latitudes due to rapid warming of the Arctic region (Francis and Vavrus, 2015; Kidston et al., 2015; Nakamura and Huang, 2018). Thus, there is increasing evidence that anthropogenic greenhouse gas emissions are involved in more frequent atmospheric blockings exacerbating extreme weather patterns (Mann et al., 2017; Nabizadeh et al., 2019), and that they may contribute to generating much climate instability in the future.

This global change is expected to induce a spatial mismatch between locally adapted plant and tree species populations and the optimal climatic conditions to which they have historically adapted, leading potentially to local maladaptation (Andalo et al., 2005; Aitken and Whitlock, 2013; Aitken and Bemmels, 2016; Saenz-Romero et al., 2020). Assisted population migration (AM), which is a human-assisted movement of seed sources to locations expected to harbor future climatic conditions like those at their geographical location of origin, has been proposed to minimize maladaptation of future local populations (Andalo et al., 2005; Aitken and Whitlock, 2013). Hence, AM has been adopted (with current implementation in some jurisdictions in Canada) as a proactive adaptation strategy to maintain forest productivity and reduce the vulnerability of forest ecosystems to climate change (Beaulieu and Rainville, 2005; Pedlar et al., 2011, 2012; Aitken and Whitlock, 2013).

Universal response and transfer functions have been developed over the last decades to guide movement of seed sources in the context of climate change (e.g., Rehfeldt et al., 1999; Wang et al., 2010; Pedlar et al., 2021). However, most of these models were based on distances in average temperature and precipitation between the local seed source and plantation locations. Given the increasing climate instability, AM based solely on modeling average shifts in optimal climatic envelops likely do not take account of possible maladaptation to increasingly more extreme and unstable local climate conditions (Benito-Garzón et al., 2013b; Park and Talbot, 2018; Benomar et al., 2022). For instance, late-spring frost which occurs after bud burst or shoot flush has been reported to cause severe damage to tree species in forest stands and plantations in temperate and boreal regions (Reichstein et al., 2013; Vitasse et al., 2019; Zohner et al., 2020; Lamichhane, 2021). The risk of frost damage implies uncertainty in survival and yield expectations from boreal forest ecosystems in the event of climatic warming (Aitken and Bemmels, 2016; Ma et al., 2019; Frelich et al., 2021; Lamichhane, 2021).

Vulnerability to late-spring frost may be influenced by seed source and local adaptation, and frost tolerance remains an important consideration when implementing seed transfers designed to mitigate harmful effects of climate change (Aitken and Whitlock, 2013; Benito-Garzón et al., 2013a; Marquis et al., 2020; Benomar et al., 2022). The survival rate and performance of seed sources when transferred to sites that are currently colder but where the temperature will increase over time due to climate change may be compromised by frost damage. Malmqvist et al. (2018) found, for instance, that coastal (warmer origin) seed sources of Douglas-fir (*Pseudotsuga menziesii*) were severely and frequently damaged by late-spring frost compared to interior seed sources (colder origin) when growing in colder plantation sites in Sweden (northern site). Moreover, coastal seed sources had a lower survival rate than interior sources.

In eastern continental Canada, southern populations of white spruce (*Picea glauca* [Moench] Voss) had a higher probability of damage by spring frost than northern ones given their earlier bud break (Marquis et al., 2020). Inversely, late-spring frost damages in Norway spruce (*Picea abies* [L.] Karst) were more pronounced in northern seed sources than southern sources after six growing seasons in comparative forest plantations in Sweden (Svystun et al., 2021). Similar results were reported for lodgepole pine (*Pinus contorta* Dougl. Ex Loud. var *latifolia* Engelm.) in a large trial in Canada (Montwé et al., 2018). These adverse effects are expected to be even more pervasive under anticipated conditions of earlier and warming spring conditions causing earlier shoot flush and growth accumulation (Beaulieu et al., 2004; Man and Lu, 2010; Owens et al., 2011).

The objectives of this study were to document and quantify the consequences of an early spring shoot flush followed by a late-spring frost and its ensuing extensive damages in a young white spruce reciprocal transplant trial related to testing for assisted migration. In doing so, we wanted to determine the relationships between the levels of frost damage and frost intensity, tree height, and seed source origin (SO), and how these results might impact AM strategies.

## Materials and Methods

### Genetic Material and Plantation Sites

**FIGURE 1:**
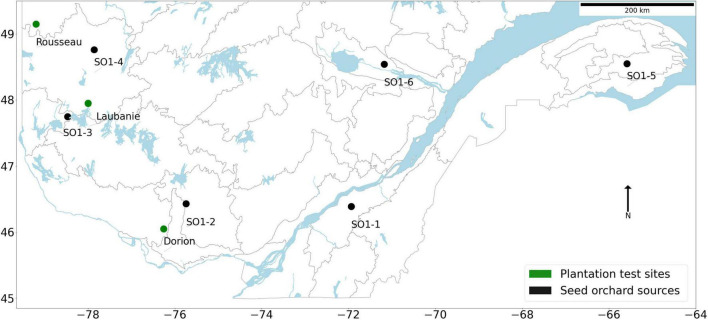
Location of the three plantation test sites (green circles) and their local seed sources, and the six seed sources (black squares) replicated on each test site. The plantations were established in Spring of 2015.

The white spruce seed sources used in this study originate from six first-generation local seed orchards (SO) established by the Ministère des Forêts, de la Faune et des Parcs of Quebec (MFFP) and commonly used for reforestation in Quebec, Canada, and from three local seed sources, used as controls and corresponding to the three study sites (Figure 1). The first-generation SO were established about 30 years ago separately for each region using phenotypically selected plus-trees from local natural forests (Figure 1). For two consecutive years (2008 and 2009), open-pollinated seeds were collected in each SO. After mixing the collection years for each seed source, seedlings were produced in the MFFP forest nursery at St-Modeste Quebec, Canada (47.50°N, 69.23°W) following standard nursery cultural practices for Quebec (Lamhamedi et al., 2006; Villeneuve et al., 2016).

The study area is located in the eastern Canadian boreal/temperate forest under northern mid-latitudes. Three forest sites were surveyed for this study, Dorion, Laubanie, and Rousseau, each separated by hundreds of km (Figure 1). These sites, respectively, represent the southern, central, and northern parts of the white spruce commercial zone in the western region of the boreal/temperate forest of Quebec. Natural forest stands harvested in 2012 were previously covering the Dorion and Rousseau sites. The Laubanie site was formerly occupied by black spruce (*Picea mariana* [Mill.] B.S.P.) plantations harvested in 2012. The reciprocal transplant trial was established by MFFP in spring 2015 on these three sites using a randomized complete block design with four blocks. In each plantation site, each block was partitioned into seven plots within which the seven sources (six SO sources and the local source) were assigned randomly. The size of each plot was about 730 m^2^ and contained 144 trees (12 by 12 rows of trees). Two-year-old seedlings were planted at densities of 2,000 stems ha^–1^. More details regarding seed sources and plantation sites have been provided (Benomar et al., 2016, 2018; Villeneuve et al., 2016). The tested seed sources were also previously shown to harbor genetic differentiation in growth-related traits recorded at earlier ages, thus reflecting the existence of local genetic adaptation (Villeneuve et al., 2016; Otis Prud’homme et al., 2018), in agreement with earlier common garden studies in eastern white spruce (Li et al., 1997).

### Climatic Data

Daily maximum (*T*_*max*_) and minimum (*T*_*min*_) temperatures during the seven growing seasons since field test establishment (2015–2021) were recorded for each of the three plantation sites using climate data from the nearest weather station available on the Government of Canada database^1^. The nearest stations (Wright, Val d’Or, Lac Berry) were 23, 25, 75 km far from the Dorion, Laubanie, and Rousseau sites, respectively. The growing degree days at a reference temperature of 5°C (GDD5) were calculated as:


$$\left\{ \begin{array}{c} \mathrm{if}\;T_{\text{max}}+T_{\text{min}}\geq 5\;\overset{}{\Rightarrow }\;\text{GDD}\,5=\sum_{i=1}^{i=365}\frac{T_{\text{max}}+T_{\text{min}}}{2}-5\\ \text{if}\;T_{\text{max}}+T_{\min}<5\;\overset{}{\Rightarrow }\;\text{GDD}\,5=0 \end{array}\right.$$

### Late Frost Damage Assessment

The late-spring frost events were recognized by days with minimum temperature below −2°C, that is, the *T*_*min*_ at which cells start to freeze (Bigras and Hébert, 1996; Bigras et al., 2004). The frost events occurred in the last week of May 2021, and frost damage recognized as pink to brown and dying of newly emerged shoots were confirmed 1 week later following the frost events in the three sites. The observed damages affected only new growth, confirming that the frost occurred after bud flush and the beginning of shoot elongation in all sites. The observations were made on 64 trees within each plot (64 trees × 4 blocks × 7 seed sources × 3 sites) for a total of 5,376 trees. The scoring of trees with frost damage and the severity of damage were assessed 2, 3, and 4 weeks following the frost event in Rousseau (northern site), Laubanie (central site) and Dorion (southern site), respectively.

**FIGURE 2:**
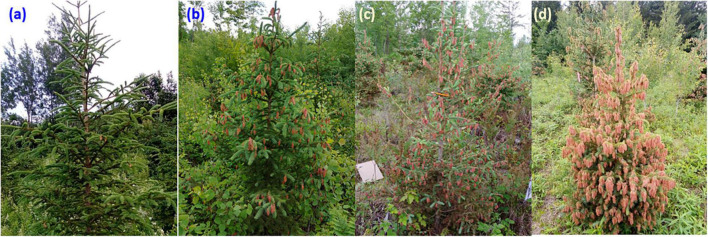
Classification of 2021 late-spring frost damage severity: trees with **(a)** no damage, **(b)** low damage, **(c)** moderate damage, and **(d)** severe damage.

For each tree, the severity of frost damage was quantified visually based on the proportion of burned shoots. Four classes (levels) of frost damage severity were defined, the first (no damage) corresponded to trees with very insignificant or without frost damage (Figure 2a), the second (low) corresponded to trees with at most one-third of damaged shoots (Figure 2b), the third (moderate) corresponded to trees with at most two-third of damaged shoots (Figure 2c), and the fourth (severe) corresponded to trees with over two-third of damaged shoots (Figure 2d). The same observer assessed frost damage in all sites to minimize evaluation bias. Given that almost no trees were observed without frost damage on either site, only the three last classes of frost damage severity were considered in statistical analyses (see below).

### Tree Height Measurements

Tree height was measured after six and seven growing seasons (2020 and 2021) on the 64 central trees in each plot for a total of 5,376 trees (64 trees × 4 blocks × 7 seed sources × 3 sites). The 2020 tree height was measured at the beginning of the 2021 growing season in June after the frost events. Total height at the end of the 2021 growing season was also measured after bud set and growth cessation in September. Growth cancelation rate was estimated as the proportion of trees with no current year height growth among live trees.

### Statistical Analyses

Analyses were performed in python (v.3.9) using the python *statsmodels* package (Seabold and Perktold, 2010). Frost damage and growth cancelation rates were analyzed using binomial logistic regressions using the site, the latitude of seed origin and their interactions as predictors. The effects of sites, seed sources and their interactions on tree height data and the proportions of trees with frost damage by class of damage severity (low, moderate, and severe) were assessed using linear models. The proportions of lowly, moderately, and severely damaged trees were arcsine transformed and tree height data was log transformed to satisfy model homoscedasticity. Linear regression analyses were carried out to examine the relationship between tree height, frost intensity (*T*_*min*_) and the proportion of severely damaged trees. As the height of the trees varied greatly between plantation sites, a covariance analysis was used to assess the effect of the site on the slope and intercept of the relationship between tree height and the proportion of severely damaged trees.

## Results

### Spring Climate Conditions and Late Frost Intensity

**FIGURE 3:**
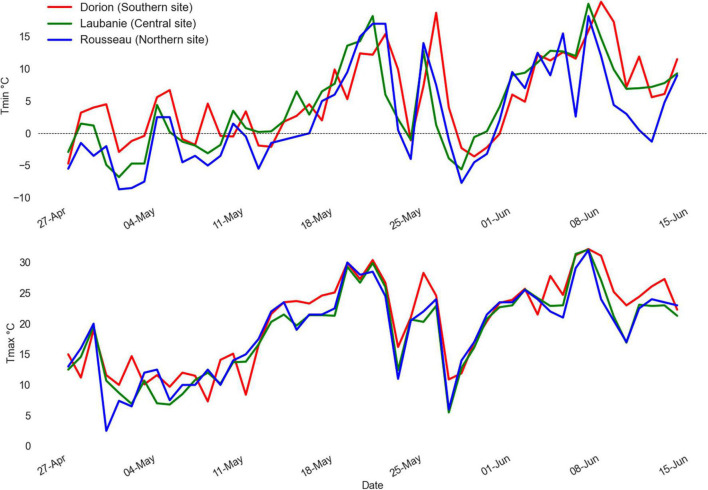
Daily maximum and minimum temperatures recorded at the nearest weather station from May 1st to June 15th 2021 for the three plantation sites.

During spring 2021, daily maximum (*T*_*max*_) and minimum (*T*_*min*_) temperatures increased linearly for mid-April to reach a max of 15 and 30°C for *T*_*min*_ and *T*_*max*_, respectively, at the end of the third week of May (Figure 3). Following this period, a decrease in both *T*_*max*_ and *T*_*min*_ during the last week of May marked by two consecutive frost events (*T*_*min*_ ≤ −2°C). The first event lasted 1 day (May 24), and the second lasted 4 days (May 27–30). During the second event, the *T*_*min*_ reached −7.7, −5.6, and −3.6°C in Rousseau (northern), Laubanie (central), and Dorion (southern) plantations, respectively, (Figure 3), indicating that the frost episode was intense for both the northern and central plantation sites. *T*_*min*_ and *T*_*max*_ were both lower in the northern site, followed by central and southern sites (Figure 3).

**FIGURE 4:**
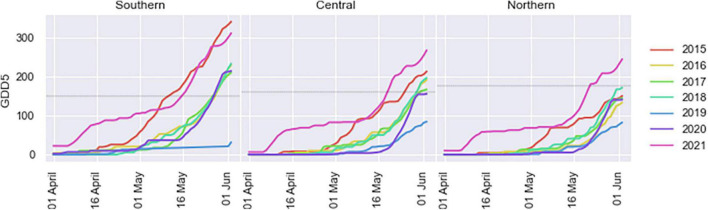
Cumulative growing degree days at 5°C (GDD5) during spring at each plantation test site during their seven growing seasons. Dashed horizontal line represents the GDD5 corresponding to the predicted average date of bud break date at each test site based on Marquis et al. (2020) for the northern site and Li et al. (1993) for the southern site.

Among the seven growing seasons experienced in the three test plantations (from 2015 to 2021), the climate conditions for 2015 and 2021 growing seasons were characterized by a warmer spring, leading to an early reach of the GDD5 threshold (between 150 and 175 degree days) triggering early bud flush in all sites (Figure 4). At the same time, these 2 years were characterized by severe frost events following bud bursts based on the corresponding GDD5 threshold (Figures 3, 4).

### Levels of Late Frost Damage Among Test Sites, Seed Sources, and According to Tree Height

**FIGURE 5:**
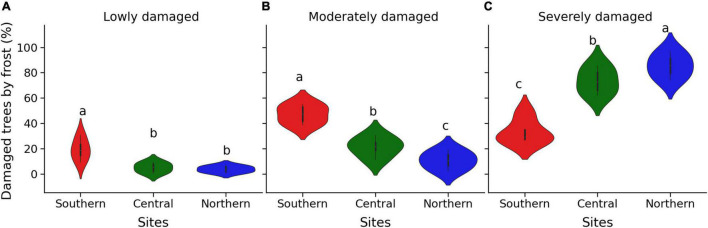
Violin plots of the rate of damaged trees (%) during the late-spring frost of 2021 in the three plantation test sites. **(A)** Lowly damaged, **(B)** moderately damaged, and **(C)** severely damaged. The violin plot combines a box plot and density plot. The box ends indicate the upper (third) to lower (first) quartiles of the value ranges, and the whiskers indicate the highest and lowest observations. Means with the same letter indicate no significant differences with *P* ≥ 0.05.

**FIGURE 6:**
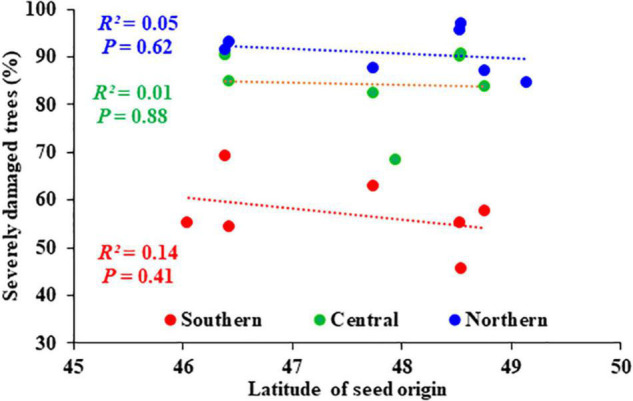
Relationship between the proportion of damaged trees by the late-spring frost of 2021 and the latitude of seed origins (*n* = 7 that is, the 6 tested seed sources across sites and the local seed source at each site) in the three plantation tested sites; (Southern, Central, Northern). Regression lines were not significant with *P* ≥ 0.05.

**FIGURE 7:**
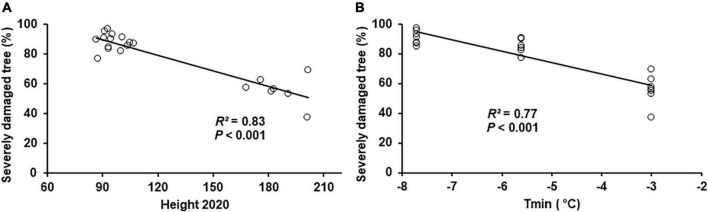
Relationships between the proportion of severely damaged trees by the late-spring frost of 2021 and **(A)** tree height before the late-spring frost event and **(B)** frost intensity.

The frost damage rate was similar among plantation test sites and among seed sources (Table 1), and it was 99.8% on average, such that almost all trees were affected by frost damage. However, the proportion of trees by class of frost damage severity differed among sites (*P* = 0.001) but not among seed sources, including the local provenances (Table 1). In addition, the interaction between sites and seed sources was not significant (Table 1), indicating that the seed sources responded similarly to different frost intensities on all sites. The percentage of trees with severe frost damage increased from the southern to the northern site (Figure 5), and inversely the percentage of trees with low or moderate frost damage decreased from the southern to the northern site (Figure 5). The proportion of severely damaged trees was unrelated to the latitude of seed origin irrespective of the plantation sites (Figure 6). The proportion of severely damaged trees was linearly and inversely related to tree height before the frost episode (Figure 7), and the slope of the relationship was similar among the three plantation sites. Similarly, the proportion of severely damaged trees was linearly and inversely related to plantation minimum temperature (*T*_*min*_) during the late most severe frost event.

**TABLE 1:** Results of analysis of variance for the rate of frost-damaged trees, the rate by class of damage severity, tree height, and growth cancelation rate for the various ANOVA effects (sites, seed sources, and the site × seed source interaction), degrees of freedom (df), *F* values, and associated probabilities (*P*).

df	Sites		Seed sources		Sites × Seed sources	
	2		6		12	
	*F*	*P*	*F*	*P*	*F*	*P*
Rate of frost-damaged trees	0.38	0.68	0.42	0.85	0.43	0.94
**Rate by class of damage severity**						
- Low	17.49	0.0010	0.81	0.48	0.85	0.40
- Moderate	31.06	<0.0001	0.59	0.80	0.25	0.98
- Severe	57.92	<0.0001	1.16	0.60	1.02	0.30
2020 tree height	2,935.37	<0.0001	12.77	<0.0001	9.89	<0.0001
2021 tree height	3,131.20	<0.0001	12.90	<0.0001	13.64	<0.0001
2021 growth cancelation rate	76.14	<0.0001	1.86	0.36	0.96	0.32

### Growth Cancelation Rate and Tree Height Variation Among Seed Sources

**FIGURE 8:**
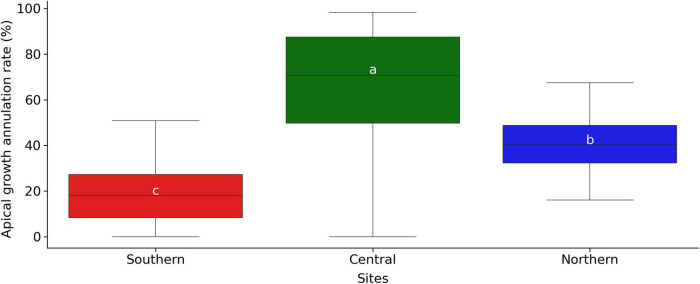
Annual growth cancelation rate (%) after the late-spring frost of 2021 in the three plantation test sites. The horizontal line inside the boxes marks the median for the observations, the box ends indicate the upper (third) to lower (first) quartiles of the value ranges, and the whiskers indicate the highest and lowest observations. Means with the same letter indicate no significant differences with *P* ≥ 0.05.

Growth cancelation rate due to the late frost episodes was significantly higher in the central site (Laubanie), followed by the northern (Rousseau) and the southern (Dorion) sites (Figure 8). However, growth cancelation rate was not significantly different among seed sources (Table 1). On the other hand, significant variation in tree height at the end of the 2020 and 2021 growing seasons was observed among seed sources (Table 1), indicating the existence of local adaptation for cumulative height growth since the establishment of test plantations in spring of 2015 and thus, prior to the late frost events of May 2021.

## Discussion

A shift in spring phenology in northern ecosystems due to winter and spring warming has been documented for a recent 30-year period (1982–2012), and it is expected to increase under future climate (Liu et al., 2018; Lamichhane, 2021). On the other hand, late-spring frost frequency and intensity may increase due to atmospheric blocking phenomena (Francis and Vavrus, 2015; Mann et al., 2017; Nabizadeh et al., 2019), thus resulting in a compound effect with spring warming. The vulnerability of plant species to frost events may vary and despite the existence of adaptive genetic variation within spruce species (e.g. Li et al., 1997; Beaulieu et al., 2004), adaptation limits can be exceeded depending of the tree phenological status when the frost event occurs, such as what we observed in the late spring of 2021.

### Early Warm Spring Growing Conditions

According to the National Oceanic and Atmospheric Administration, 2021 was world’s sixth warmest year on record. Although, 2019 and 2020 were also among the three warmest years on Earth, it is in 2021 that our study plantations experienced their warmest winter and spring (Figure 4). The temperatures observed then were also above the normal for the last 30 years in eastern Canada. These exceptionally early warm spring conditions favored a quick accumulation of growing degree-days at 5°C (GDD5), allowing to reach very early the minimum threshold needed to trigger bud burst and shoot elongation (Figures 2, 4) and thus, causing an earlier initiation of the growing season in all of our white spruce test plantations, which were separated by many hundreds of kilometers.

### Late-Spring Frost Intensity and Severity of Damage

The end of May late-spring frost event that occurred in 2021 was harsh in the central (−5.6°C) and northern (−7.7°C) plantation sites, and moderate (−3.6°C) in the southern plantation site, and the intensity of the frost had a direct incidence on its severity of effects (Figure 7). Frost damage severity was related positively to frost intensity and negatively to tree height before the late frost event (Figure 7). Microsite conditions and landscape attributes such as elevation, slope shape, and angular slope were reported to influence frost severity (Marquis et al., 2021). Based on our results, it appears that taller trees (around 2 m) escaped some damages, given that layers of very cold air are likely to be located closer to the ground, thus affecting more severely smaller trees. Thus, it likely that with trees gaining in age and height, the levels of frost damage from such severe cold spell should decrease, given that much of their crowns would become less exposed to such intense frost.

### Frost Damage and Spring Phenology

The effect of late-spring frost on growth cancelation and long-term growth patterns has received little attention so far, and only indirect assessments from frost-ring data are available (Marquis et al., 2020). In the present study, growth cancelation rate was higher in the central plantation (74%), followed by northern (46%) and southern (22%) plantations. The late-spring frost occurred about 10 days after the average threshold GDD5 across sites for bud burst (Figures 3, 4). However, the accumulation of GDD5 was different among sites. Given its more northern and colder spring climate, the northern site accumulated GGD5 later than the central site, and much later than the southern site during the warm spring of 2021 (Figure 4). Also, given that bud burst occurs in a gradient from the base to the top of the tree (Dhont et al., 2010), we may expect that most apical buds in trees established on the northern site were still at the initial stage of bud burst during the frost event.

Therefore, the counterintuitive moderate growth cancelation rate observed in the northern plantation test, as compared to the central and southern sites, may have resulted from the later bursting of apical buds and thus, more restricted shoot elongation (Bigras and Hébert, 1996; Bigras et al., 2004) at the time of the severe late-spring frost. This would have resulted in less damage to newly emerged shoots at the northern site. Conversely, it should not be surprising that growth cancelation rate was highest for the central plantation test (Figure 8) where a combination of early shoot elongation from earlier accumulation of GDG5 compared to the northern site, and more intense frost episode as compared to the most southern site, would have contributed to the more severe growth cancelation rate observed in this central site. Thus, this site illustrates well the combined detrimental effects of early warm spring phenology and late intense frost on canceling current year growth.

### Genetic Variation Among Seed Sources for Levels of Late-Spring Frost Damage

No significant variation among seed sources was detected in this study with respect to levels of late-spring frost damage or for 2021 growth cancelation rate (Table 1 and Figure 6). However, the level of frost damage varied among trees within seed sources, which could be related to variation in relation to GDD5 needed to trigger bud flush and frost vulnerability, in addition to possible heterogenous microsite conditions and within seed source variation in tree height, as reported above. Also, the low and moderately damaged trees observed within each seed source may have resulted from an asynchronous breaking of buds within trees and consequently, a partial avoidance of frost damage. Thus, in the future, we expect to observe a significant negative correlation between the recovery capacity of the trees and the severity of frost damage.

Given the generally severe effects of this late-spring frost after an early warm spring where trees had well initiated their annual shoot growth, a lack of local genetic adaptation for such a severe frost stress should not be surprising. The late cold spell was less intense for the most meridional site but even there, given that trees had initiated their annual growth earlier due to earlier spring warming conditions, almost no trees could escape frost damages. On this site, even if northern seed sources haboured less severely damaged trees on average (Figure 6), which could reflect a signature of local adaptation in relation to later bud flush and less elongated new shoots, this trend was not significant. In relation to this, Falconer and Mackay (1996) indicated that the largest variation among genetic elements, such as seed sources, is most commonly observed at a 50% incidence rate for threshold traits. Lower incidence may result in distinctive variation for only very sensitive elements while higher incidence would largely overtake the threshold in a quite uniform fashion among elements, for instance due to physiological incapacity of elongating new shoots to resist well such frost intense. In the present case, we were likely well over such an intermediate level and it is likely that for such a high stress, little meaningful genetic variation had evolved among seed sources regarding local adaptation.

For several tree species, northern seed sources were reported to be more sensitive to late-spring frost as they usually require fewer GDD5 (growing degree-days at 5°C) to flush their buds than the southern or coastal seed sources generally more adapted to warmer climates (Blum, 1988; Li et al., 1997; Lesser and Parker, 2004; Søgaard et al., 2008; Rossi, 2015; Marquis et al., 2020). For other species, the inverse relationship was reported (Beuker, 1994; Malmqvist et al., 2018; Montwé et al., 2018; Svystun et al., 2021), implicating that the general trend remains uncertain. Indeed, genetic differentiation may exist but of reduced nature, or it could be that the different patterns observed could result from yearly variation in timing and rate of accumulation of GDD5. In the present study, seed sources suffered similarly from frost damage along the tested climate gradient (Figure 6), indicating that their phenological stage was quite uniform at the time of late-spring frost, and that they may have responded to similar heat unit accumulation (GDD5) for bud break, as previously reported for white spruce (Li et al., 1993; Lu and Man, 2011; Villeneuve et al., 2016).

Also, in an earlier common garden study of the phenology of young white spruce families from diverse geographical origins spanning a large part of the natural distribution in eastern Canada (Li et al., 1993, 1997), while much genetic variation was found for bud set timing among provenances and families within provenances, much reduced genetic variation was observed for bud burst when the spring accumulation of GDD5 was quite abrupt, which is reminiscent of the early spring warming conditions witnessed in the present study before the late-spring frost of end of May 2021. Our results implicate that in the general context of temperature warming with increasing climate instability, such conditions of early spring warming followed by late-frost effects are likely to be more frequent, for which limited adaptive genetic variation would be available for tree breeders to select for more tolerant seed sources to such extreme stress.

### Recovery Capacity of Trees From Extreme Stress

Given the various levels of frost damage suffered by trees, some differences among trees within seed sources are expected to be seen in their recovery capacity. In line with this, we also expect to observe variation among seed sources in their recovery capacity, given the observed variation in tree height among seed sources after the 2020 and 2021 growing seasons, and as previously observed for more juvenile traits related to growth in this long-term reciprocal transplant experiment (Benomar et al., 2016; Villeneuve et al., 2016; Otis Prud’homme et al., 2018). Also, genetic variation in recovery from severe drought stress was observed among provenances and families in white spruce grown in a common garden (Depardieu et al., 2020). Genetic correlations were positive between cumulative height growth and the recovery capacity of young white spruce families after a severe drought stress experienced 23 years after the establishment of this common garden study (Depardieu et al., 2021), and in another common garden study established on two different sites, 10 and 12 years, respectively, after their establishment (Laverdière et al., 2021). Such a trend at the genetic level indicate a positive relationship between lifespan tree vigor and the recovery capacity of trees at their juvenile stage.

We thus anticipate that, on average, seed sources with superior tree vigor will better recover from this late cold spell stress if enough time is given for recovery without suffering repeatedly from other severe stress. Accordingly, recovery will be monitored in the following years in the plantation tests. But it is also possible that with increasing climate instability over continental mid-latitudes, recovery may be disturbed by other climate-related stress such as drought episodes, which are seemingly increasing in frequency and intensity under northern mid-latitudes (Depardieu et al., 2020; Laverdière et al., 2021).

### Implications for Assisted Migration and Seed Transfer Modeling

Our results show the drastic influence of late-spring frost damage on tree growth of the current year, with no differences related to local adaptation within the geographical limits of our study. Our results also follow the recent findings of the important roles of repeated spring and fall frosts in the observed growth stagnation of white spruce plantations in the boreal mixedwood region of Quebec (Marquis et al., 2021). Therefore, more attention should be paid to the occurrence of such frost episodes and other climate anomalies in forest plantation management and seed transfer modeling. The use of multivariate seed transfer models will have to integrate the effects of such extreme climate anomalies if variation in recovery capacity exists among seed sources, as suggested above.

Based on our results, the northward transfer of southern seed sources was not associated with more frost damage given that for the three test plantation sites, all seed sources were damaged similarly between each other including local seed sources. Thus, we may expect a lack of trade-off between productivity and frost resistance in white spruce at a regional scale, at least for the large mid-latitude region surveyed in the present study and in the context of intense late-spring frosts. Nevertheless, we could only assess physical damage to shoots of the current year. Investigations regarding the levels of frost damage in cambial cells and alteration to xylem physiology, and about potential genetic variation in capacity and time to recovery among the tested seed sources, are thus needed to reduce uncertainties regarding seed transfer modeling. Also, it would be advisable to examine how the various seed sources would react to early autumn frost events, as there appears to be more genetic variation related to the timing of bud set and cold conditioning (Li et al., 1993).

### Implications for Plantation Programs and Genetic Testing

If frost injuries to young spruce plantations are expected to become more frequent with increasing climate instability, ways to reduce severe damage must be sought. For instance, aspen cover has been shown to provide frost protection to white spruce at the young age in mixed stands (Man and Lieffers, 1999; Filipescu and Comeau, 2011). Multi-site testing and monitoring of such multi-specific plantation schemes over many years with appropriate controls would be necessary to measure the likely reductions in growth productivity that would result from more competition for water and light (Jobidon et al., 2003; Macadam and Kabzems, 2006; Benomar et al., 2013), and to determine the optimal timing of plantation release from the protective cover. Even more, such potential stagnation of height growth could increase the vulnerability of young trees to frost damage by extending the time period of great sensitivity of young trees to late-spring frosts. Indeed, such detrimental effects could be exacerbated during drought episodes (Shovon et al., 2021), which are also on the rise with climate change under northern mid-latitudes. Thus, in examining alternative silvicultural systems, a right balance should be sought between survival rate, longer period of vulnerability to stress, and growth potential.

Also, in common garden studies of conifers under northern mi-latitudes where juvenile growth is usually an objective of testing, competition for light or water from a protective broad-leaf tree cover may introduce an estimation bias in evaluating genetic variation within and among seed sources, as well as for other traits negatively or positively correlated to juvenile growth. Given the increasing climate instability and frequency of severe stress episodes, ways of establishing common garden studies and genetic testing may thus need to be re-assessed if the establishment of plantations under protective tree cover is more broadly implemented.

## Conclusion and Perspectives

Climate change impacts ecosystem dynamics and functioning through changes in mean climatic conditions and climate anomalies associated with extreme events. Late-winter and early spring warmings that could be followed by acute cold spells have increased in the last decade and will likely continue throughout the mid-latitudes of the northern hemisphere, mainly as a result of more frequent atmospheric blocking events. Here, we assessed damage caused by a succession of two such anomalies, an early spring warming followed by a late-spring frost that occurred in 2021 in three white spruce test plantations separated by many hundreds of kilometers. Irrespective of frost intensity, our results showed a similar response of seed sources (including local provenances) to late frost events in terms of frost damage severity and growth cancelation. The lack of local adaptation to such extreme climate events resulted largely from limited genetic differentiation in the white spruce material tested, in relation to early and most likely synchronized bud flush stimulated by early spring warming conditions.

The severity of frost damage was positively related to frost intensity and negatively related to tree height. Thus, we may expect trees to be less vulnerable to late-spring frost as they grow and gain height. Forest managers should thus pay more attention to frost tolerance at the early age of trees when establishing forest plantations, and evaluate possible ways of establishing plantations under temporary protective cover without affecting too much juvenile growth, which should be a challenging task.

Within the limits of our study, the results also showed that a northerly transfer of southern seed sources is not necessarily associated with a higher risk of late-spring frost damage, though an adverse relationship may have been detected under a less intense late-spring frost. In the context of post-stress recovery, AM could still be largely relying on the optimal climate envelope of seed sources, because it should optimize lifespan tree vigor and the recovery capacity of affected trees. However, more investigations regarding the amount of genetic variation in the post-stress recovery capacity of trees and their seed sources are needed to firmly support this recommendation.

## Data Availability Statement

The raw data supporting the conclusions of this article will be made available by the authors, without undue reservation.

## Author Contributions

LB set the study design, collected data, conducted statistical analyses, and drafted the manuscript. JBo participated to study design, drafting the manuscript, and obtaining funds. MP participated in obtaining funds, providing support and access to plantation tests, and reviewed and improved the manuscript. JBe participated in obtaining funds and helped drafting the manuscript. ML participated to study design, data collection, and drafting the manuscript and funding of this project. All authors contributed to the article and approved the submitted version.

## Conflict of Interest

The authors declare that the research was conducted in the absence of any commercial or financial relationships that could be construed as a potential conflict of interest.

## Publisher’s Note

All claims expressed in this article are solely those of the authors and do not necessarily represent those of their affiliated organizations, or those of the publisher, the editors and the reviewers. Any product that may be evaluated in this article, or claim that may be made by its manufacturer, is not guaranteed or endorsed by the publisher.

## Footnotes

1
https://climat.meteo.gc.ca/index_f.html
